# Does dexmedetomidine have an antiarrhythmic effect on cardiac patients? A meta-analysis of randomized controlled trials

**DOI:** 10.1371/journal.pone.0193303

**Published:** 2018-03-01

**Authors:** Xiaoyan Ling, Hongmei Zhou, Yunjian Ni, Cheng Wu, Caijun Zhang, Zhipeng Zhu

**Affiliations:** 1 Outpatient Nursing Department, the Second Affiliated Hospital of Jiaxing University, Jiaxing City, Zhejiang Province, China; 2 Department of Anesthesiology, the Second Affiliated Hospital of Jiaxing University, Jiaxing City, Zhejiang Province, China; University of Tampere, FINLAND

## Abstract

**Background:**

Cardiac surgery patients often experience several types of tachyarrhythmias after admission to the intensive care unit (ICU), which increases mortality and morbidity. Dexmedetomidine (DEX) is a popular medicine used for sedation in the ICU, and its other pharmacological characteristics are gradually being uncovered.

**Purpose:**

To determine whether DEX has an antiarrhythmic effect after cardiac surgery.

**Methods:**

The three primary databases MEDLINE, Embase (OVID SP) and the Cochrane Central Register of Controlled Trials (CENTRAL) were searched, and all English-language and randomized control-designed clinical publications comparing DEX to control medicines for sedation after elective cardiac surgery were included. Two colleagues independently extracted the data and performed other quality assessments. A subgroup analysis was performed according to the different medicines used and whether cardiopulmonary bypass (CPB) was applied. All tachyarrhythmias that occurred in the atria and ventricles were analyzed.

**Results:**

A total of 1295 patients in 9 studies met the selection criteria among 2587 studies that were screened. After quantitative synthesis, our results revealed that the DEX group was associated with a lower incidence of ventricular arrhythmia (VA, OR 0.24, 95% CI 0.09–0.64, I^2^ = 0%, P = 0.005) than the control group. Subgroup analysis did not reveal a significant difference between the DEX and propofol subgroups (OR 0.13, 95% CI 0.03–0.56, I^2^ = 0%, P = 0.007). Additionally, no difference in the incidence of atrial fibrillation (AF) was observed regardless of the different control medicines (OR 0.82, 95% CI 0.60–1.10, I^2^ = 25%, P = 0.19) or whether CPB was applied.

**Conclusions:**

This meta-analysis revealed that DEX has an antiarrhythmic effect that decreases the incidence of VA compared to other drugs used for sedation following cardiac surgery. DEX may not have an effect on AF, but cautious interpretation should be exercised due to high heterogeneity.

## Introduction

The incidence of complications has decisive significance for overall mortality in patients following cardiac operations. A high incidence of complications is directly correlated with increased hospitalization and economic costs as well as with the quality of life of the patients. Postoperative arrhythmia (POA) is a type of complication that can occur following cardiac surgery. Atrial tachyarrhythmia is a common postoperative heart rhythm disorder and includes the so-called postoperative atrial fibrillation (POAF). Ventricular arrhythmia (VA) is also a major complication with lower frequency and less clarity.

Increasing evidence suggests that POAs are mainly caused by the diversity of patient characteristics and surgery items [1–2]. Currently, increasing studies have focused on congenital heart disease in infants, for whom postoperative arrhythmias frequently occur in the early postoperative period [3–5]. According to reports, the incidences of different types of arrhythmias can reach 48% in pediatric cardiac patients [6–7]. Moreover, up to 40–50% of patients will develop POAF during hospitalization following cardiac surgery [8]. The occurrence of atrial fibrillation (AF) not only prolongs hospitalization but also increases the cost [9–10]. Furthermore, treatment of these arrhythmias is limited by ineffective antiarrhythmic therapies and the significant adverse effects of drugs. The development of antiarrhythmic drugs has always been challenging and limited.

Dexmedetomidine (DEX) is a highly selective α-2 receptor agonist that has been applied safely and efficiently in perioperative cardiac surgery since its approval by the U.S. Food and Drug Administration [11]. Since that time, DEX has been popular for cardiac surgery patients on fast-track anesthesia when recovery is required during the ICU stay [12–14]. Based on several randomized control studies, DEX can provide safe and effective sedation [15–16], facilitate extubation [15], and reduce delirium [17–18], AF [19], and renal [20] and myocardial injury [21]. Meta-analyses and reviews have also indicated that this drug is safe and efficacious for post-cardiac surgery patients [22–23].

Ettema and his coworkers hypothesized that DEX was necessary to prevent postoperative complications from preadmission interventions for older cardiac surgery patients [24]. Based on their theory, rate and rhythm control strategies are typically the focus for supraventricular tachyarrhythmias, and prompt cardioversion for VAs is necessary [1]. Prophylactic drugs, such as beta-blockers [25], amiodarone [26] and lidocaine[27], have their own limitations, and there is controversy regarding the effectiveness of antiarrhythmia medications. As a first-line sedation medicine [28], DEX seems to be a promising option for postoperative cardiac patients, but clinical and review articles have reported conflicting results on its efficiency as an arrhythmia treatment following infant heart surgery [29–31], and few studies have reported its arrhythmia treatment efficiency in adult cardiac patients, with the exception of Liu’s report [19]. Nevertheless, efforts must be taken to prevent POAs, and the lack of relevant reviews on the use of DEX as a treatment for POAs is noteworthy.

## Methods

### Search strategy

Two investigators independently searched the computerized databases Embase (OVID SP), the Cochrane Central Register of Controlled Trials (CENTRAL), and MEDLINE. All eligible articles written in English between 1966 and May 2017 were chosen. The search strategy included the words “Dexmedetomidine”, “Adrenergic alpha-Agonists”, “Precedex”, “Thoracic Surgery”, “Cardiac Surgical Procedures”, “cardiac surgery”, “Arrhythmias, Cardiac” and “Anti-Arrhythmia Agents”. Various combinations of these free words were also used S1 File. The search concluded in June 2017, and 2 new interesting reports were identified for further review.

### Eligible studies

All eligible studies met the following conditions: 1, all randomized controlled trials involving valve replacement surgery or coronary artery bypass surgery with or without cardiopulmonary bypass that compared DEX to control drugs; 2, sedation time <24 h in the ICU regardless of when extubation occurred; 3, the patients were older than 18 years; 4, there were no limitations on the time of drug infusion and dose limit; and 5, reported the incidence of AF or VA.

### Data extraction and quality control

After the full-text articles were retrieved, two investigators independently conducted quality assessments of the articles using the Cochrane Risk Bias Assessment Tool of the Cochrane Handbook for Systematic Reviews of Interventions (Higgins 2011) [32]. The assessment items included the following: random sequence generation, blinding of participants and personnel, blinding of outcome assessment, incomplete outcome data, allocation concealment, selective reporting, and other biases S1 and S9 Tables. Next, the data were extracted, and a third operator intervened when disagreements occurred. The primary outcomes were the incidence of AF and ventricular tachyarrhythmia (including ventricular fibrillation (VF) and ventricular tachycardia (VT)) S10 and S11 Tables. The GRADE system for each outcome was applied to evaluate the quality of the grade.

### Statistical analysis

Review manager (version 5.3) [33] was used to pool and analyze the eligible studies when two or more studies reported the outcomes of interest. The fixed-effects model was applied throughout the whole analysis. A random-effects model was used only in the presence of clinically and statistically significant heterogeneity (P>0.1, I^2^<50%). The odds ratio (OR) was chosen as the effect measure for dichotomous outcomes. Subgroup analyses were performed based on the different infused medicines and whether cardiopulmonary bypass (CPB) was utilized. Sensitivity analyses were also performed by removing studies with low quality or a small sample size to confirm the stability of the results after analysis. After meta-analysis of all the outcomes, the summaries of the findings for each outcome were created with the GRADE system to evaluate the quality of the evidence.

## Results

### Included studies

**Fig 1:**
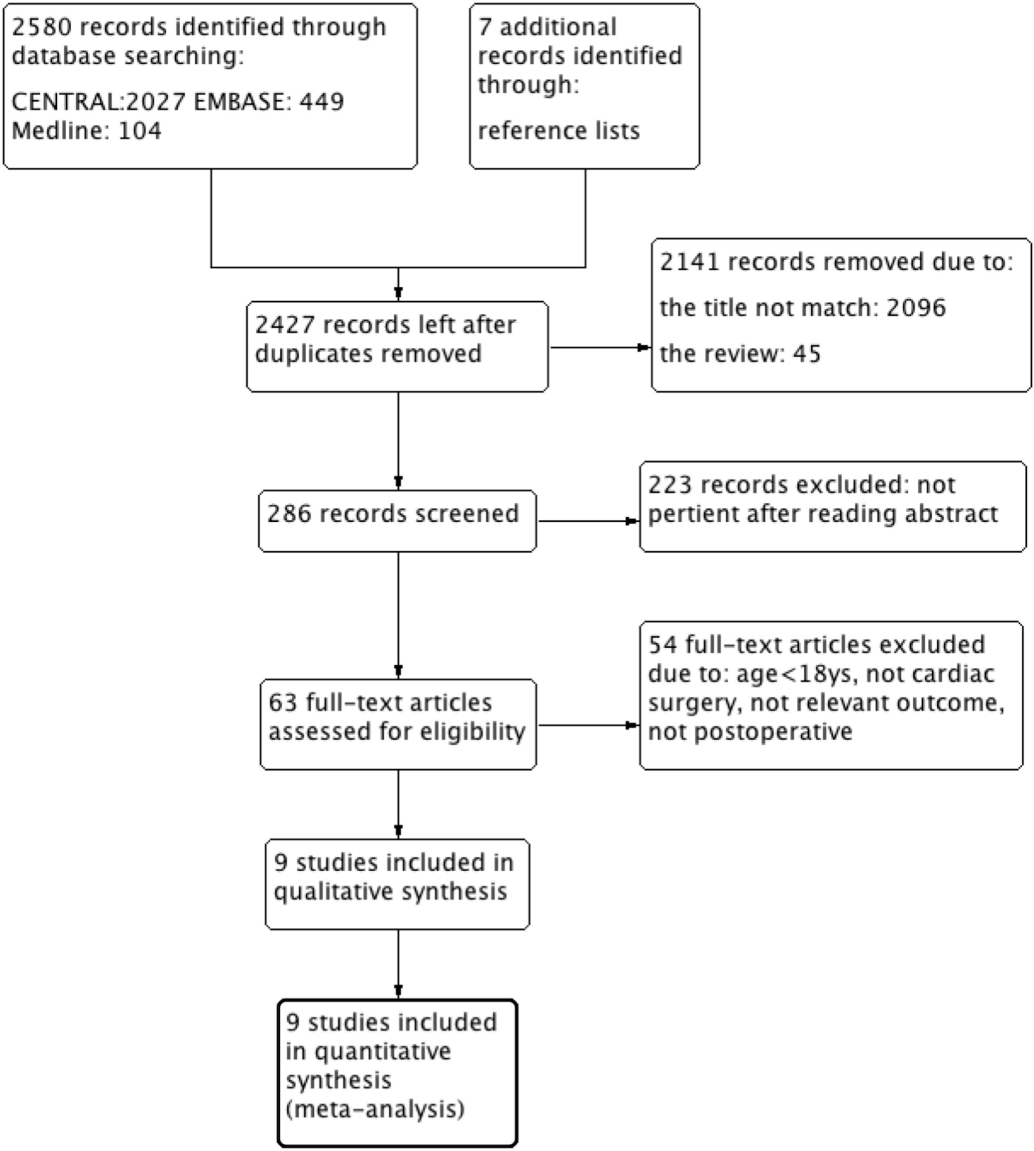
Study flow diagram.

Twenty-five hundred and eighty records were identified in the electronic databases Medline (104), EMBASE (449) and CENTRAL (2027). After checking for duplications, screening the titles or abstracts and reviewing the content, 63 full-text articles were retrieved from a database purchased by our own unit. Fifty-four articles were excluded due to various reasons. Finally, the remaining 9 studies were included in the quantitative analysis [15–21, 34–35] (Fig 1).

### Basic characteristics of the studies

Nine studies were included in this review. The total number of patients was 1295, and the included patients across all studies ranged from 11 to 152 Table 1. There were 7 studies (449 in the DEX group, 461 in the control group) that compared DEX to propofol [15–17, 19, 21, 34–35] and accounted for approximately 70% of the total patients. One study compared DEX to morphine [18] and placebo [20]. Five studies performed cardiac surgery without CPB assistance [15, 16, 20–21, 35]. Intravenous infusion of DEX began primarily upon admission to the ICU in 6 studies. Only 1 study adopted a low infusion rate (0.04 μg/kg/h) without a bolus volume upon admission to the ICU [20].

**Table 1:** Characteristics of the included studies.

Author (publication year)	Age	Patient number	Surgery type	DEX intervention time	DEX intervention	Control infusion
Herr(2003)	61.9	295	CABG	Sternal closure~24 h in the ICU	1.0 μg/kg induction then maintained by 0.2 to 0.7μg/kg/h	Propofol used, but no detailed data
Corbett(2005)	63.6	89	CABG	ICU admission~1 hr postextubation	1 μg/kg induction then maintained by 0.4 μg/kg/h.	Propofol: 0.2 to 0.7 μg/kg/h
Shehabi(2009)	71.5	306	On-pump cardiac surgery*	ICU admission~extubation/leaving the ICU/48 h maximum	0.1 to 0.7 μg/kg/h	Morphine: 10 to 70 μg/kg/h
Goksedef(2013)	58	100	CABG	ICU admission~24 h maximum	0.04 μg/kg/h	Placebo
Ren(2013)	60	162	CABG	Vascular anastomosis grafting~12 h in the ICU	0.2–0.5 μg/kg/h	Propofol: 2–4 mg/kg/h
Karaman(2015)	62.5	64	CABG	ICU admission~extubation	0.2–1.0 μg/kg/h	Propofol: 1.0–3.0 mg/kg/h
George(2016)	72.7	183	On-pump cardiac surgery*	ICU admission~extubation	0.4 μg/kg bolus followed by 0.2 to 0.7 μg/kg/h	Propofol: 25 to 50 μg/kg/min
Liu(2016)	62.5	90	On-pump cardiac surgery*	ICU admission~extubation	0.2–1.5 μg/kg/h	Propofol: 0.3–3 mg/kg/h
Liu(2017)	53	61	On-pump cardiac surgery*	ICU admission~extubation	0.2–1.5 μg/kg/h	Propofol: 5 to 50 μg/kg/min

CABG, coronary artery bypass grafting; ICU, intensive care unit; DEX, dexmedetomidine; and

*, on-pump cardiac surgery including valve surgery and/or CABG.

### Risk of bias

According to the Cochrane handbook, a high risk of bias was present in less than 25% of the included studies. Following a detailed evaluation of each study, we determined that 4 studies in this review could be considered as high quality because a high risk of bias was not identified [16–18,35]. Three studies were considered to be of moderate quality due to incomplete data that resulted in disproportionate data loss [20–21,34]; for example, one study mentioned “respiratory function” indicators in the methods, but no results were provided in the subsequent sections [20]. The remaining 2 studies were classified as low quality due to high risk of bias from either the lack of randomization, attrition balance or ITT [15,19].

### Summary of findings by the GRADE system

Using the GRADE system, two main outcomes were graded: AF was graded as low-quality evidence because of incomplete data, disproportionate data loss [15, 19] and imprecision bias due to a small number of studies with wide confidence intervals. Ventricular tachyarrhythmia was graded as moderate-quality evidence because only one level was downgraded due to methodological bias [21, 35] Table 2.

**Table 2:** Summary of findings for each outcome.

Outcomes	Illustrative comparative risks (95% CI)		Relative effect	No of participants	Quality of the evidence	Comments
	Assumed risk Control drugs	Corresponding risk DEX	(95% CI)	(Studies)	(GRADE)	
	Study population					
Atrial fibrillation	206 per 1000	169 per 1000 (124 to 227)	RR 0.82 (0.6 to 1.1)	1295 (9 studies)	⊕⊕⊝⊝ low^1^^,^^2^	
	Moderate					
Ventricular tachyarrhythmia	45 per 1000	11 per 1000 (4 to 29)	RR 0.24 (0.09 to 0.64)	845 (4 studies)	⊕⊕⊕⊝ moderate^3^	
	Moderate					

**CI:** confidence interval; **RR:** risk ratio; DEX, dexmedetomidine

^1^, attrition bias occurred due to incomplete data or NO ITT

^2^, several low number of studies with very wide confidence intervals; and

^3^_,_ several methodological limitations occurred, such as random distribution and double blinding.

### What is the influence of DEX on VA?

**Fig 2:**
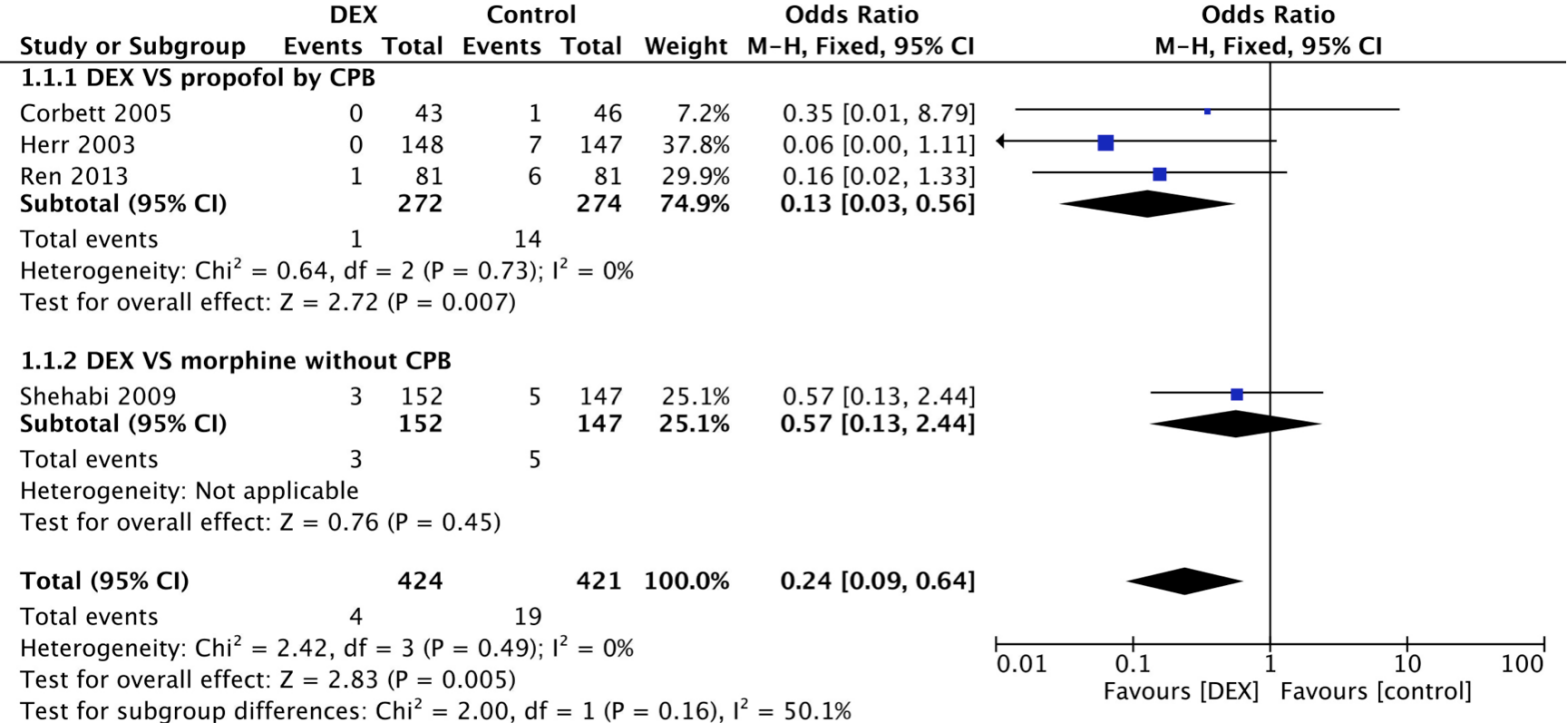
Forest plot of the incidence of ventricular arrhythmia after cardiac surgery with sedation by DEX compared to control medicine.

Four of the 9 included studies, with 1295 participants, evaluated VA. The overall outcome of the four studies revealed a lower incidence of VA in the DEX (1%) group than in the control (5%) group (P = 0.005). Of these studies, 3 were pooled together in the meta-analysis as they compared DEX with propofol. A statistically significant difference in the incidence of VA was revealed between the DEX and propofol subgroups (OR 0.13, 95% CI 0.03–0.56, I^2^ = 0%, P = 0.007). One study, which was not pooled due to the lack of CPB [18], exhibited a lower incidence of VA in the DEX group (2%) than in the morphine group (3%) (Fig 2).

### What is the influence of DEX on AF?

**Fig 3:**
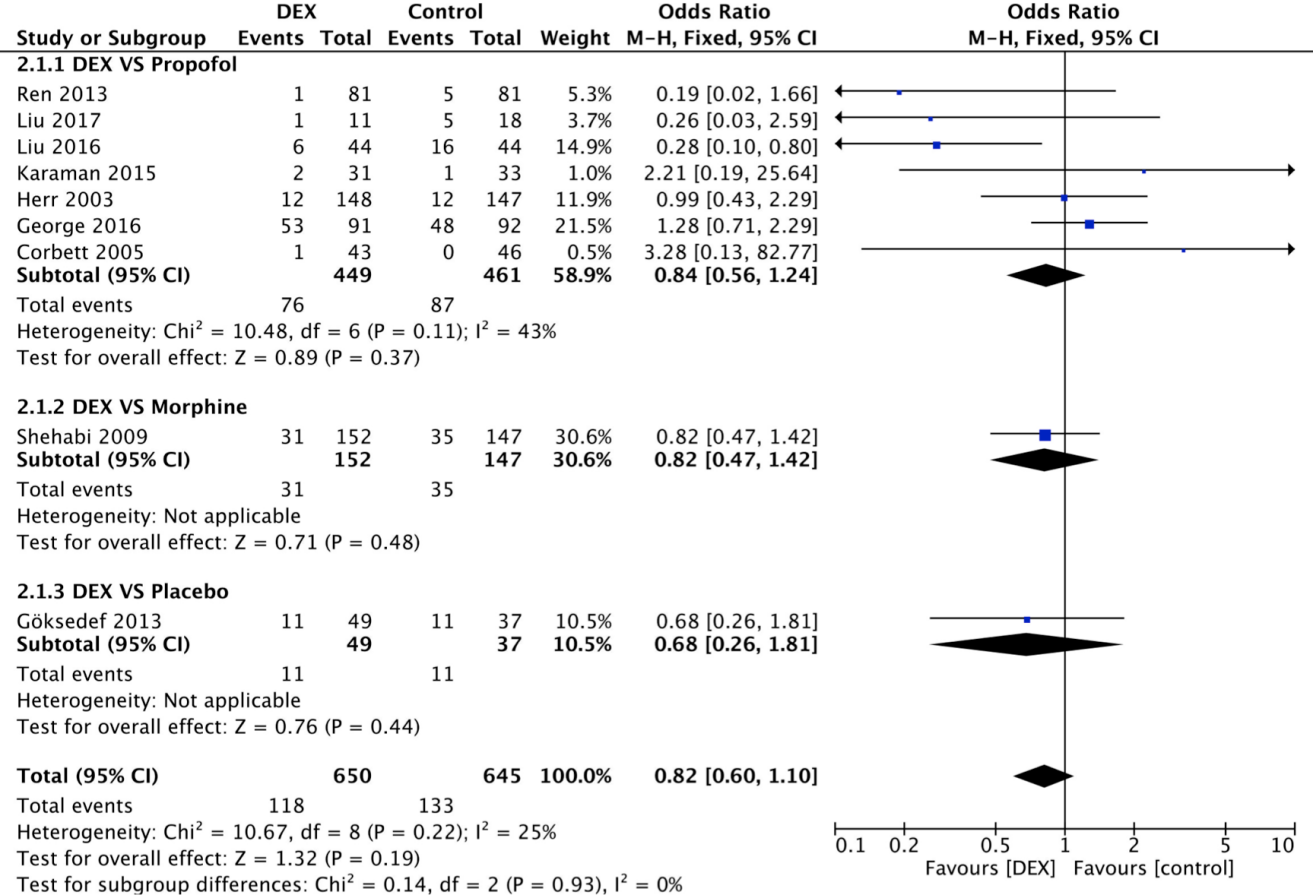
Forest plot of the incidence of AF after cardiac surgery with sedation by DEX compared to different medicines and the subgroup analysis.

**Fig 4:**
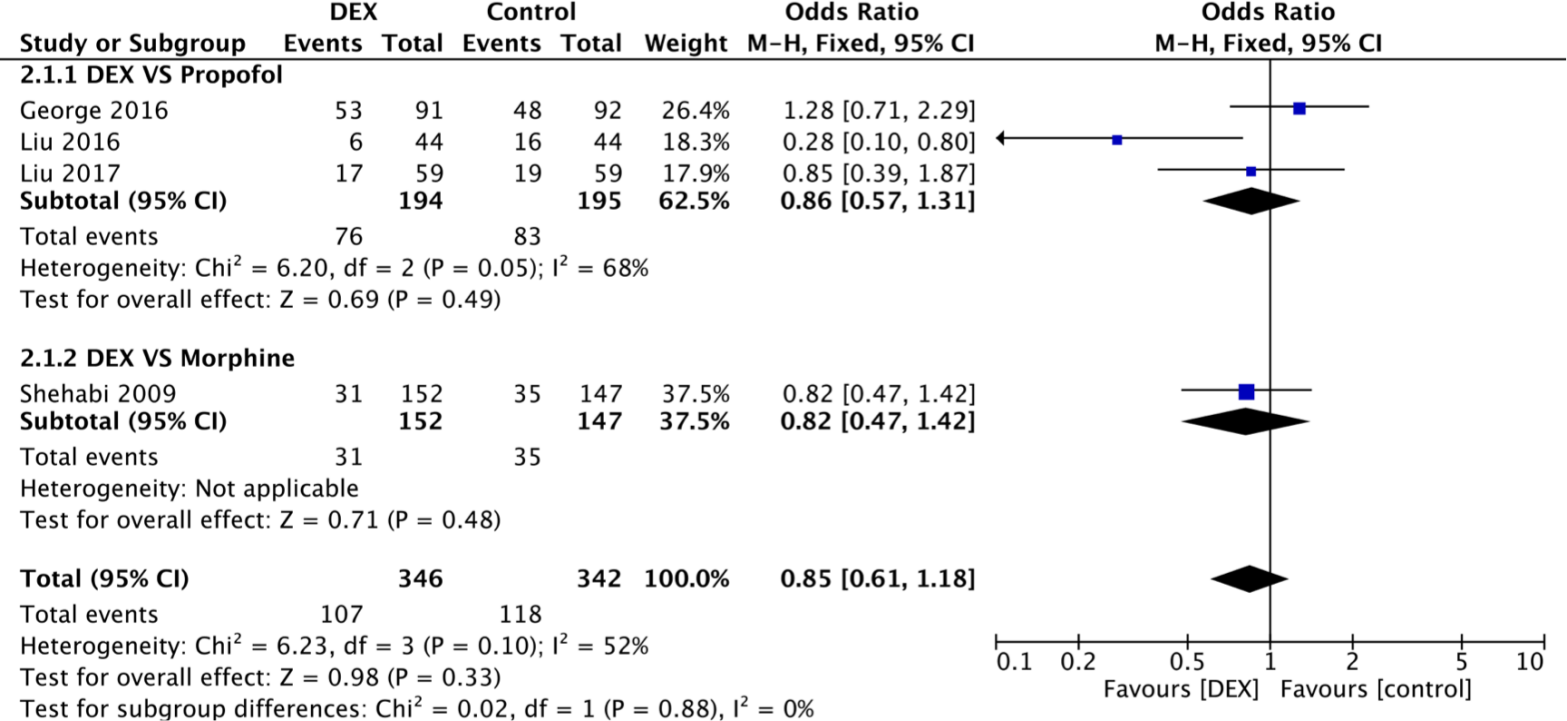
Forest plot of the incidence of AF among patients after cardiac surgery under CPB with sedation by DEX compared to control medicine and the subgroup analysis.

**Fig 5:**
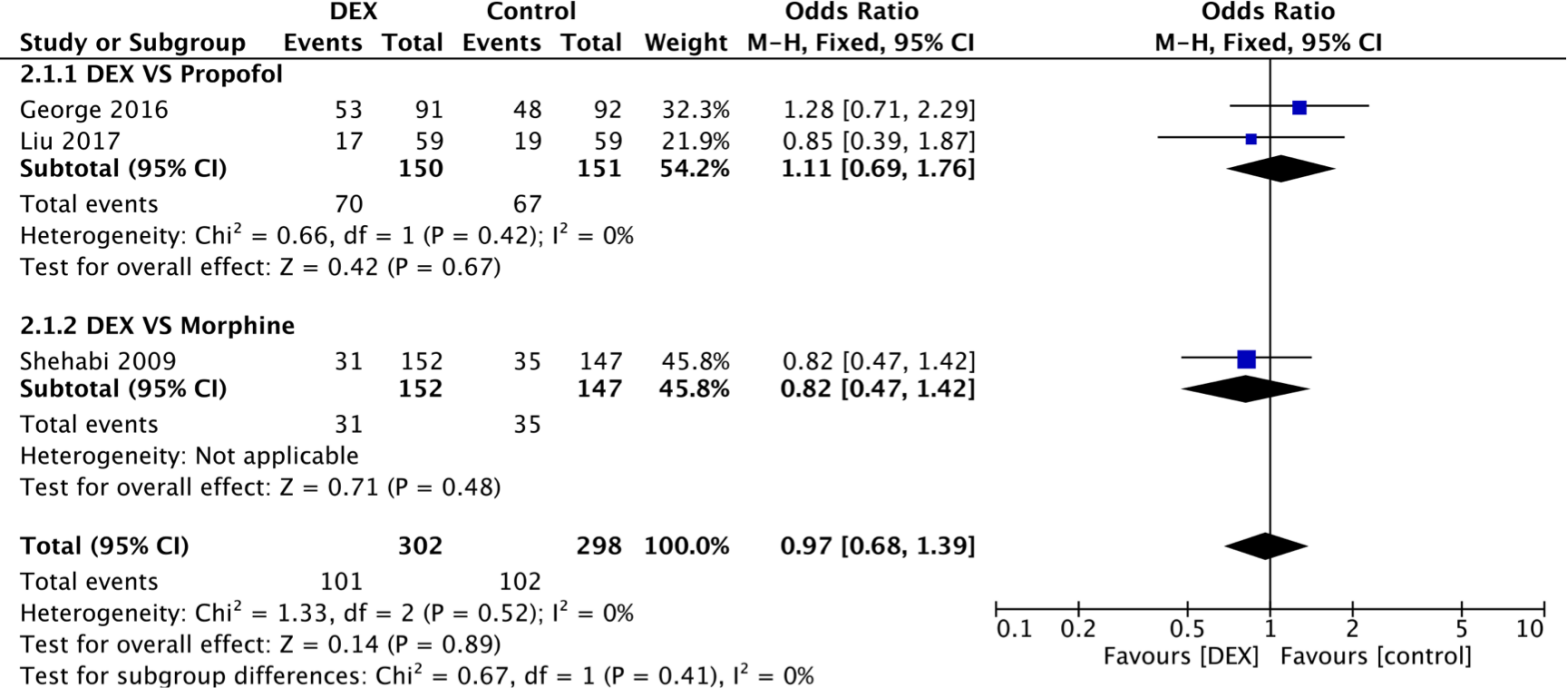
Forest plot of the sensitivity analysis of the incidence of AF among patients after cardiac surgery under CPB with sedation by DEX compared to control medicine.

**Fig 6:**
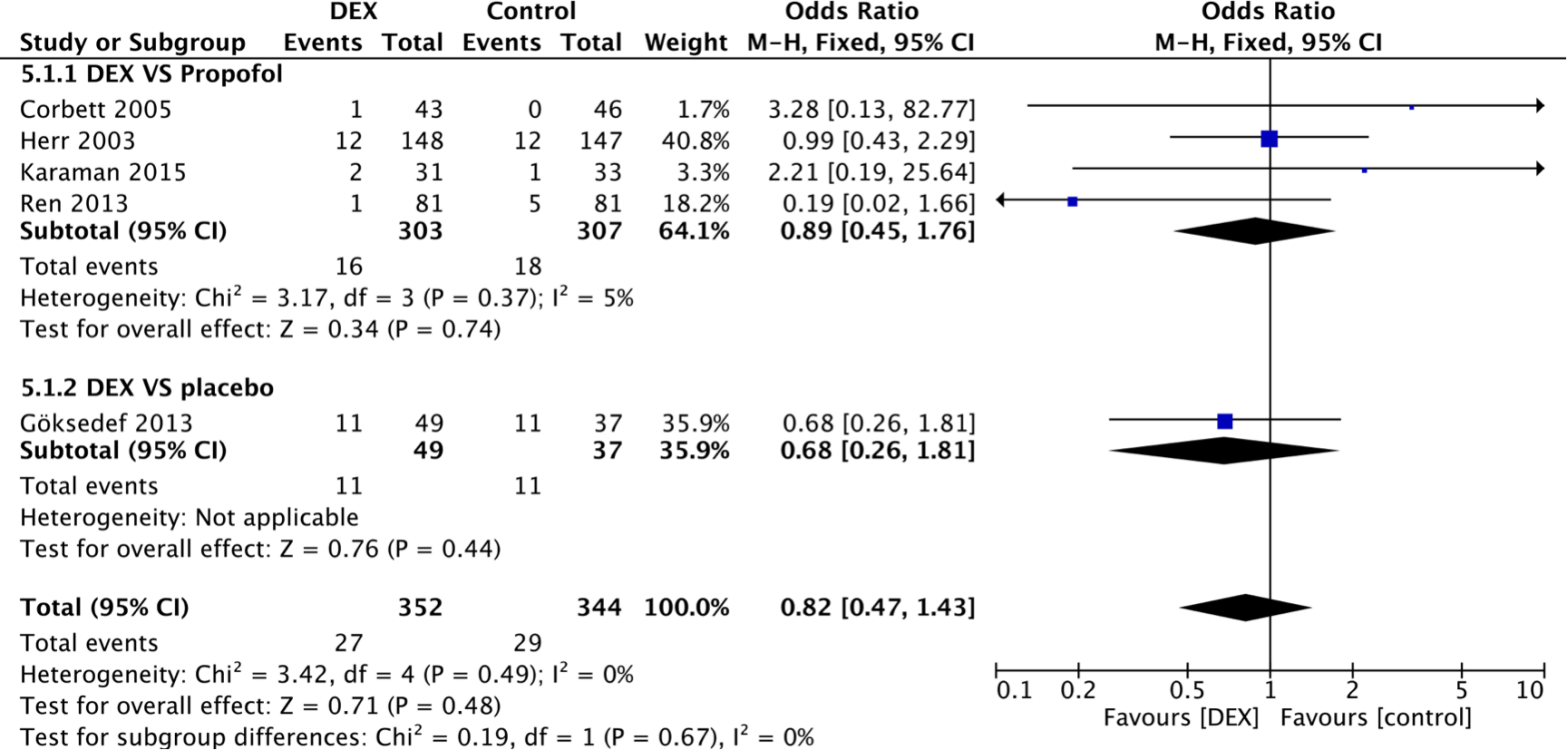
Forest plot of the incidence of AF among patients after cardiac surgery without CPB with sedation by DEX compared to control medicine and the subgroup analysis.

The incidence of AF is presented in all 9 studies. In the medicine control subgroup, no reduction in the overall incidence of AF was observed (P = 0.19) (Fig 3). Seven studies involving propofol use were compared in the subgroup analysis, and similar results were found (OR 0.84, 95% CI 0.56–1.24, I^2^ = 43%, P = 0.37). In the CPB subgroup, the incidence of AF was presented in 4 studies, three of which compared DEX with propofol (Fig 4). A random-effects model was adopted due to high heterogeneity (I^2^ = 68%). No significant difference was identified between the DEX and propofol subgroups (OR 1.11, 95% CI 0.69–1.77, I^2^ = 0%, P = 0.67), even after one low-quality study was removed for sensitivity analysis [19] (Fig 5). Moreover, in the without-CPB subgroup, no significant differences were identified among the main comparisons between DEX and propofol (4 studies, 610 patients) (OR 0.89, 95% CI 0.45–1.76, I^2^ = 5%, P = 0.74) (Fig 6).

## Discussion

With the popularity of DEX sedation for ICU patients, an increasing number of functions and shortcomings for this drug have been reported. Moreover, the controversial reports urgently need to be resolved. Our current meta-analysis indicates that infusion with DEX following cardiac surgery may decrease the incidence of VT but not AF compared with control drugs. Currently, the pathogenesis of POAs is largely understood. The proposed risk factors for POAs include hypoxia, ischemia, trauma, inflammation, catecholamines and electrolyte abnormalities, but some patients have different characteristics [36]. It is well known that tachyarrhythmias can lead to reduced diastolic filling time and cardiac output, which can result in myocardial ischemia and hypotension. Related research has indicated that the excitation of sympathetic nerves is the primary pathogenesis of tachyarrhythmia following cardiac surgery [37]. AF following cardiac surgery is a thoroughly studied postoperative tachyarrhythmia [38–42]. Specifically, new-onset AF is the focus of current research studies [39]. According to Banach’s meta-analysis, the incidence of AF among patients after cardiac surgery doubles the risk of death [43]. The ACCP Guidelines for the Prevention and Management of Postoperative Atrial Fibrillation After Cardiac Surgery were proposed by The American College of Chest Physicians in 2005 [44]. The recommendations for the management of POAF were subsequently updated in 2016 [45]. The potential mechanism underlying POAF may be activation of a systemic proinflammatory state, myocardial irritation, and heightened sympathetic tone. Although various functional drugs are recommended, it remains unclear whether these drugs have definitive effects. Another arrhythmia that can lead to sudden cardiac death in the cardiac ICU is VT or VF, which has an incidence of 5–8% [46]. It has been reported that VT or VF after cardiac surgery not only worsens long-term prognosis but also increases in-hospital mortality [47–48]. There are many risk factors for VT/VF, including ischemia, sympathetic stimulation, and electrolyte abnormalities [49]. However, there are not many medications available for the treatment and prevention of VT or VF nor many related clinical research studies. Amiodarone may currently be the most effective drug [50], but clinical research for the development of new drugs is urgently required.

DEX is a highly selective new-generation alpha-2 adrenergic receptor agonist and has been applied safely for sedation. DEX also exhibits anxiolytic, analgesic, and sympatholytic properties [51]. By activating G-protein transmembrane alpha-2 receptors in the brain, DEX can theoretically influence the transmission of sympathetic activity from the central to the peripheral nervous system and elicit an antiarrhythmic effect. This anti-epinephrine effect has already been shown to be effective by Hayashi’s research [52]. Activation of the vagus nerve was also subsequently thought to be a mechanism responsible for the antidysrhythmic effect of DEX [53]. Additionally, studies have reported the multifunctional characteristics of DEX treatment in cardiac surgery, which include reducing myocardial ischemia-reperfusion injury [21, 54] and inhibiting the inflammatory response [55–56]. In conclusion, all of the above features indicate that DEX has antiarrhythmic effects. Additionally, these effects were confirmed for the first time in a cohort study showing that DEX can decrease the incidence of ventricular and supraventricular tachyarrhythmias in patients following congenital heart surgery [31].

To our knowledge, this is the first meta-analysis of the effects of DEX on ventricular tachyarrhythmias in adult cardiac patients. Our results revealed, with moderate-quality evidence Table 2, that DEX can decrease the overall incidence of VT and VF by 76% compared to control drugs. However, few RCTs have investigated this relationship in adult patients; most related studies are review articles or have focused on pediatric patients [30–31]. Nevertheless, the results of these studies are consistent with those of our study. Additional prospective RCTs are required to verify the antiarrhythmic effect of DEX. In contrast, prophylactics for AF are a controversial topic in different types of patients; Ai and his coworkers reported the ineffectiveness of DEX for lung cancer patients [57], but Liu’s study reported positive effects of DEX in the prevention of AF [19]. Some related reviews have also strived to clarify AF risk factors and drug prevention strategies [3, 45]. Our meta-analysis concluded that DEX did not exhibit effective antiarrhythmic qualities compared to control drugs (P = 0.56). Moreover, this study included evidence that was graded to be of low quality Table 2. When targeting different control drugs that were formulated ahead of time, the same statistical results were found (P = 0.84, 0.68, and 0.82, respectively). However, the subgroup analysis by CPB exhibited obvious heterogeneity (I^2^ = 59%), which was eliminated after sensitivity analysis with the removal of one study [19]; subsequently, consistent results were obtained. The heterogeneity from the removed study was probably attributed to the young age of the study cohort (approximately 50 years) and the shortened clamping time (approximately 50 minutes), which were different from the other studies. Increased age and clamping times are well-known risk factors for POAF [58–60] and directly influence the incidence of AF and patient prognosis. However, given the multifactorial influences on AF and the low quality of evidence, this part of the results should be interpreted cautiously.

Several limitations are present in our meta-analysis: 1, Due to the shortage of RCTs, only 1 study was evaluated in the subgroup analysis according to the different drugs infused, which can lead to incapability when quantitatively merging the results. 2. All of the collected outcome measures were not the main outcomes of the included studies; therefore, it is possible that the tests had inadequate power. 3. Many design differences among these studies made it difficult to reduce clinical heterogeneity; for example, the timing of dexmedetomidine administration, the duration of the dexmedetomidine infusion, the presence or lack of a loading dose, and the infusion drug dosage varied.

## Conclusions

Based on this meta-analysis, we conclude that DEX elicits antiarrhythmic effects by decreasing the incidence of VA compared with control drugs following cardiac surgery. No statistically significant difference in AF incidence was observed between the DEX and control groups, but cautious interpretation should be exercised when CPB is utilized. Additional larger-scale prospective studies or further subgroup analyses are warranted in the future.

## Supporting information

S1 File Search strategy of the present study. (DOCX)

S2 File PRISMA checklist of the present study. (DOC)

S1 Table Risk of bias in Corbett’s study. (DOCX)

S2 Table Risk of bias in Djaiani’s study. (DOCX)

S3 Table Risk of bias in Herr’s study. (DOCX)

S4 Table Risk of bias in Karaman’s study. (DOCX)

S5 Table Risk of bias in Liu’s 2016 study. (DOCX)

S6 Table Risk of bias in Liu’s 2017 study. (DOCX)

S7 Table Risk of bias in Ren’s study. (DOCX)

S8 Table Risk of bias in Shehabi’s study. (DOCX)

S9 Table Risk of bias in Goksedef’s study. (DOCX)

S10 Table Primary data for AF incidence. (XLSX)

S11 Table Primary data for VA incidence. (XLSX)
