# Supplementary material for: Does dexmedetomidine have an antiarrhythmic effect on cardiac patients? A meta-analysis of randomized controlled trials
Source: PLoS One. 2018 Mar 1;13(3):e0193303. doi: 10.1371/journal.pone.0193303 (PMC5832237; doi:10.1371/journal.pone.0193303)
Supplement: S1 File — (DOCX) [file pone.0193303.s001.docx]

One searching example for medline:

#1 "Dexmedetomidine"[Mesh]

#2 "Adrenergic alpha-Agonists"[Mesh]

#3 (((Precedex [Title/Abstract]) OR Dexmedetomidin*[Title/Abstract]) OR "adren?ergic agonist*"[Title/Abstract]) OR "alpha agonist*"[Title/Abstract]

#4 #1or#2or#3

#5 "Anti-Arrhythmia Agents"[Mesh]

#6 “Arrhythmias, Cardiac”[Mesh]

#7 Antiarrhythm* or Antifibrillatory or Arrhythmi* or tachyarrhythmi* or “atrial fibrillation”

#8 #5or#6or#7

#9 "Thoracic Surgery"[Mesh]

#10 "Cardiac Surgical Procedures"[Mesh]

#11 (heart* or coronary or “coronary artery*”or cardio* or cardiac or valve* or myocardial) and (surg* or intervention* or procedure* or bypass*)

#12 #9or#10or#11

#13 #4and#8and#12
