# Supplementary material for: Does dexmedetomidine have an antiarrhythmic effect on cardiac patients? A meta-analysis of randomized controlled trials
Source: PLoS One. 2018 Mar 1;13(3):e0193303. doi: 10.1371/journal.pone.0193303 (PMC5832237; doi:10.1371/journal.pone.0193303)
Supplement: S1 Table — (DOCX) [file pone.0193303.s003.docx]

**Characteristics of included studies**：

Corbett 2005

| methods | Randomized controlled trial |
| --- | --- |
| participants | A total of 89 adult, nonemergent, coronary artery bypass graft patients with an expected length of intubation of <24 hrs. |
| Interventions | dexmedetomidine versus propofol／DEX or propofol was initiated after bypass/Patients were included if they were >18 yrs of age and required nonemergent CABG surgery with an expected MV(机械通气) length of <24 hrs. Patients were with- drawn from the study if length of intubation (LOI) exceeded 48 hrs.／／DEX (1ug/kg [actual body weight] load- ing dose intravenously administered over 15 mins, followed by a 0.4-􏰺g·kg􏰻1·hr􏰻1 intrave- nous infusion) or propofol (5-􏰺g·kg􏰻1·min􏰻1 intravenous infusion titrated within the range of 0.2– 0.7 􏰺g·kg􏰻 1 ·hr􏰻 1 or 5–75 􏰺g·kg􏰻1·min􏰻1) |
| outcomes | to assessed patient-perceived satisfaction with coronary artery bypass graft surgery after administration of DEX or propofol for intensive care unit (ICU) sedation.:   1. Treatment arms were well matched with regard to length of ventilatory support, and length of ICU stay, The overall level of sedation was significantly greater in the propofol group (p= .021), with a median (intraquartile range) Ramsay level of 4.00 (3.62 to 4.19) compared with 3.67 (3.00 to 4.15) in the DEX arm, and similar requirements for additional morphine and midazolam were observed (p = .317). 2. no significant difference was noted with regard to systolic blood pres- sure, mean arterial pressure, or the percentage of patients who developed HoTN in each group (81.4% DEX vs. 67.4% propofol, p =.132), 3. side effects：One episode each of atrial fibrillation, delirium, and drug discontinuation for oversedation occurred in the DEX treatment arm, one episode each of ventricular fibril- lation, self-extubation, pneumothorax, and delirium occurred in the propofol- treated patients. |
| notes | A total of 89 elective CABG patients participated in the study (43 DEX, 46 propofol) each group, |

***Risk of bias***

| **Bias** | **Authors’ judgement** | **Support for judgement** |
| --- | --- | --- |
| Random sequence generation (selection bias) | low risk | Qoute:Randomization to either DEX or propofol, via a random-number table, |
| Allocation concealment (selection bias) | Unclear risk | Not mentioned |
| Blinding of participants and personnel (performance bias) All outcomes | Unclear risk | Not mentioned |
| Blinding of outcome assessment (detection bias)  All outcomes | Unclear risk | Not mentioned |
| Incomplete outcome data (attrition bias) All outcomes | low risk | All patients were included in the study with no withdraw. |
| selective reporting (reporting bias) | low risk | Although No protocol;it reported all outcomes. |
