# Supplementary material for: Does dexmedetomidine have an antiarrhythmic effect on cardiac patients? A meta-analysis of randomized controlled trials
Source: PLoS One. 2018 Mar 1;13(3):e0193303. doi: 10.1371/journal.pone.0193303 (PMC5832237; doi:10.1371/journal.pone.0193303)
Supplement: S2 Table — (DOCX) [file pone.0193303.s004.docx]

**Characteristics of included studies**：

Djaiani,2016

| methods | Randomized controlled trial |
| --- | --- |
| participants | post-operative cardiac surgery patients／in patients 60 yr or older undergoing cardiac surgery. |
| Interventions | dexmedetomidine versus propofol／Upon admission to intensive care unit (ICU), patients received either dexmedetomidine (0.4 μg/kg bolus followed by 0.2 to 0.7 μg kg−1 h−1 infusion) or propofol (25 to 50 μg kg−1 min−1 infu- sion) |
| outcomes | Primary outcome was the incidence of POD(Postoperative delirium):  A.POD was present in 16 of 91 (17.5%) and 29 of 92 (31.5%) patients in dexmedetomidine and propofol groups, respectively (odds ratio, 0.46; 95% CI, 0.23 to 0.92; P = 0.028), (P<0.05)  B.POD onset,POD duration:  Median onset of POD was on postoperative day 2 (1 to 4 days) versus 1 (1 to 4 days), P = 0.027, and duration of POD 2 days (1 to 4 days) versus 3 days (1 to 5 days), P = 0.04, in dexmedetomidine and propofol groups, respectively. |
| notes | The infusion of dexmedetomidine was continued for a maximum period of 24h. If mechanical ventilation was required beyond the 24-h period, based on the institutional standard of practice, patients in the dexmedetomidine group were converted to propofol sedation.. Sedation level was assessed by using the Sedation Agitation Scale (SAS) . Pain was assessed using a standard 10-cm visual analog scale (0, no pain; 10, worst and unbearable pain). Patients received 2 mg morphine or 0.2 to 0.4 mg hydromorphone intravenously or 2 to 4mg orally if pain was 4 or more. |

***Risk of bias***

| **Bias** | **Authors’ judgement** | **Support for judgement** |
| --- | --- | --- |
| Random sequence generation (selection bias) | Low risk | Patients were randomly allocated to either dexmedetomidine or propofol (control) groups according to a computer-generated randomization code in blocks of four, aiming at subject allocation in a 1:1 ratio. |
| Allocation concealment (selection bias) | Low risk | Opaque sealed envelopes were generated according to the randomization schedule and opened by a study coordinator before surgery. |
| Blinding of participants and personnel (performance bias) All outcomes | Low risk | Single blinded |
| Blinding of outcome assessment (detection bias)  All outcomes | low risk | The primary limitation of our study was lack of blinding of the dexmedetomidine and propofol infusions. However, the testers of CAM-ICU and CAM were not aware of the study objectives. |
| Incomplete outcome data (attrition bias) All outcomes | low risk | Have incomplete data,but it has has ITT method.  A total of 185 patients were randomized and 183 analyzed. One patient died in the operating room, and one patient underwent off-pump coronary revascularization surgery based on the intraoperative decision and was excluded from the analysis. One patient who was randomized to dexmedeto- midine group received propofol sedation due to scheduling changes that resulted in unavailability of dexmedetomidine upon arrival in ICU. On the basis of a priori “intent-to-treat” analysis, |
| selective reporting (reporting bias) | Low risk | protocol available,reported all datas. |
