# Supplementary material for: Does dexmedetomidine have an antiarrhythmic effect on cardiac patients? A meta-analysis of randomized controlled trials
Source: PLoS One. 2018 Mar 1;13(3):e0193303. doi: 10.1371/journal.pone.0193303 (PMC5832237; doi:10.1371/journal.pone.0193303)
Supplement: S3 Table — (DOCX) [file pone.0193303.s005.docx]

**Characteristics of included studies**：

Herr 2003

| methods | Randomized controlled trial |
| --- | --- |
| participants | patients were adults who were scheduled for coronary artery bypass graft surgery. |
| Interventions | dexmedetomidine versus propofol／At sternal closure, patients randomized to group A received 1.0 􏰶g/kg of dexmedetomidine over 20 minutes as the loading dose, followed by a maintenance infusion of 0.4 􏰶g/kg/h. After transfer to the ICU, the infusion rate was titrated in the range of 0.2 to 0.7 􏰶g/kg/h as necessary to maintain a Ramsay sedation score26 of >3 before extu- bation. The dexmedetomidine infusions were continued for a minimum of 6 hours after extubation, up to a maximum of 24 hours total. Patients randomized to group B were started on a propofol infusion at sternal closure, No dose or rate of propofol was specified by the protocol. |
| outcomes | 1. The primary outcome was the efficacy of sedation of dexmedetomidine compared with current propofol-based ICU sedation； 2. Secondary variables were as follows: The total dose of morphine administered for pain；Time to weaning；Time to extubation；Blood pressure, heart rate, and respiratory rate were recorded。   sedation：There were no significant differences in mean Ramsay sedation scores between groups during assisted ventilation (4.5, dexmedetomidine *v* 4.7, propofol; *p* ＝0.259) (P>0.05)  there were no significant differences between the groups in median times to weaning or extubation. (P>0.05)  additional sedative/analgesic requirements：the dexmedetomidine-sedated patients required significantly less morphine than patients in the propofol group (P<0.05)。  Dexmedetomidine-sedated patients experienced an early and transient increase in mSBP of approximately 6 mmHg in the first 20 minutes after study drug start，In contrast, mSBP in the propofol group did not decrease after 30 minutes, remaining elevated approx- imately 9 mmHg above baseline until hour 16。  The incidence of treatment-emergent adverse reactions was similar between groups。  Mean heart rates were similar between groups throughout the study period。  significantly fewer dexmedetomidine patients than propofol patients required beta-blockers, high-dose diuretics (eg, lasix or furosemide), NSAIDs, or epinephrine while in the ICU。 |
| notes | Patients were randomized before surgery by sealed envelopes provided by the statistician. Investigators did not know the randomiza- tion block size. All efficacy and safety analyses presented here are for the intent-to- treat population  One limitation of this study design was that hypertension and hypotension were not specifically defined in terms of a numer-ical or percentage change in blood pressure. |

***Risk of bias***

| **Bias** | **Authors’ judgement** | **Support for judgement** |
| --- | --- | --- |
| Random sequence generation (selection bias) | Unclear risk |  |
| Allocation concealment (selection bias) | Low risk | Patients were randomized before surgery by sealed envelopes provided by the statistician. Investigators did not know the randomization block size. |
| Blinding of participants and personnel (performance bias) All outcomes | Unclear risk | Open-lable,no central computer assignment. Not mentioned |
| Blinding of outcome assessment (detection bias)  All outcomes | Unclear risk | Not mentioned |
| Incomplete outcome data (attrition bias) All outcomes | low risk | has incomplete data:  The intent-to-treat dataset consisted of 148 patients in the dexmedetomidine group and 147 in the propofol group, The proportion of the two groups is proportions |
| selective reporting (reporting bias) | Low risk | No protocol available |
