# Supplementary material for: Does dexmedetomidine have an antiarrhythmic effect on cardiac patients? A meta-analysis of randomized controlled trials
Source: PLoS One. 2018 Mar 1;13(3):e0193303. doi: 10.1371/journal.pone.0193303 (PMC5832237; doi:10.1371/journal.pone.0193303)
Supplement: S4 Table — (DOCX) [file pone.0193303.s006.docx]

**Characteristics of included studies**：

**Karaman 2015**

| methods | Randomized controlled trial |
| --- | --- |
| participants | 70 patients who underwent CABG surgery between the ages of 40–75 years and (ASA) physical condition score of <IV. |
| Interventions | The time of transfer to the ICU was regarded as the start of the study. Dexmedetomidine (0.2–1.0 μg/kg/h) and propofol (1.0–3.0 mg/kg/h) infusion doses were titrated, Infusions of dexmedetomidine and propofol were stopped with the extubation. |
| outcomes | to compare the effects of propofol and dexmedetomidine infusions on extubation times, hemodynamic and respiratory functions, complication rates and patient satisfaction scores:  A.extubation times: Group D compared to Group P (*P* < 0.001) (Fig. 2). The mean times to extubation were 265.94 ± 43.1 min for Group D and 322.52 ± 39.2 min for Group P. (P<0.05)  B.Ramsay Sedation Scores: Group P 3 (2–4) ,compared to Group D 3 (3–4)* (*P* < 0.05)  C. patient satisfaction:the median (min–max) values of Group P [7 (5−9)] were significantly lower than Group D [9 (7–10)] (*P* < 0.001).  SBP in both groups, no significant difference was observed between the groups (Fig. 3). Median heart rate was similar in both groups (Fig. 4).  no differences in the incidence of postop- erative adverse events in both groups  No significant differences between the two groups for demographic data and baseline characteristics (P>0.05) |
| notes | 64 eligible patients, Dexmedetomidine 31,propofol 33. |

***Risk of bias***

| **Bias** | **Authors’ judgement** | **Support for judgement** |
| --- | --- | --- |
| Random sequence generation (selection bias) | high risk | By using sealed envelopes, one group of patients received 0.6 μg/kg/h dexmedetomidine IV infusion (Group D) and the other group received 2 mg/kg/h propofol IV infu- sion (Group P) in a random fashion. |
| Allocation concealment (selection bias) | Unclear risk | By using sealed envelopes, |
| Blinding of participants and personnel (performance bias) All outcomes | Unclear risk | Not mentioned |
| Blinding of outcome assessment (detection bias)  All outcomes | low risk | Another limitation of our study was that the sedative drugs were not blinded due to the unique physical properties of propofol. However, we think we avoided a possible bias by evaluating patients using both BIS and RSS. |
| Incomplete outcome data (attrition bias) All outcomes | High risk | has incomplete data, Two patients were excluded during the postopera- tive period due to bleeding, a further 3 patients due to prolonged support of inotropic and vasodilating drugs, and 1 patient because of pulmonary problems, leaving 64 patients in the study.no ITT? |
| selective reporting (reporting bias) | high risk | No protocol available; “respiratory functions” was mentioned in methods,but no result. |
