# Supplementary material for: Does dexmedetomidine have an antiarrhythmic effect on cardiac patients? A meta-analysis of randomized controlled trials
Source: PLoS One. 2018 Mar 1;13(3):e0193303. doi: 10.1371/journal.pone.0193303 (PMC5832237; doi:10.1371/journal.pone.0193303)
Supplement: S5 Table — (DOCX) [file pone.0193303.s007.docx]

**Characteristics of included studies**：

*Liu 2016*

| methods | a randomized, controlled trial. |
| --- | --- |
| participants | ：age ≥18 years, elective cardiac surgery with cardiopulmonary bypass (CPB), admitted to inten- sive care unit (ICU) while intubated and ventilated, and lack of prior AF or flutter before receiving sedation in the ICU. |
| Interventions | randomized to receive either dexmedetomidine (0.2–1.5 μg/kg/h) or propofol (0.3–3 mg/kg/h) open-label titrated  。 |
| outcomes | primary endpoint was the incidence of postoperative atrial fibrillation, and the secondary end points were the length of ICU stay, length of hospital stay, and hospital costs.   1. Atrial fibrillation occurred in 6 of 44 patients (13.6 %) in the dexmedetomidine group compared to 16 of 44 patients (36.4 %) in the propofol group (odds ratio = 0.28; 95 % confidence interval, 0.10, 0.80; P = 0.025).   B.The median (interquartile range) length of ICU stay in the dexmedetomidine group was significantly lower than in the propofol group (2.9 (2.4–3.5) vs 3.5 (2.7–4.5 days, P = 0.008), The incidence of hypotension was higher in the dexmedetomidine group than in the propofol group (25/44 (56.8 %) vs 13/44 (29.5 %); P = 0.017). |
| notes |  |

***Risk of bias***

| **Bias** | **Authors’ judgement** | **Support for judgement** |
| --- | --- | --- |
| Random sequence generation (selection bias) | Low risk | The patients were randomized in a 1:1 ratio to receive sedation with either propofol (control) or dexmede- tomidine according to the random number table. |
| Allocation concealment (selection bias) | unclear risk |  |
| Blinding of participants and personnel (performance bias) All outcomes | high risk | Open-lable.Can not be performed blinded. |
| Blinding of outcome assessment (detection bias)  All outcomes | high risk | Open-lable.Can not be performed blinded. |
| Incomplete outcome data (attrition bias) All outcomes | low risk | The proportion of the two groups is proportions |
| selective reporting (reporting bias) | Low risk | has protocol available，no unavailable data. |
