# Supplementary material for: Does dexmedetomidine have an antiarrhythmic effect on cardiac patients? A meta-analysis of randomized controlled trials
Source: PLoS One. 2018 Mar 1;13(3):e0193303. doi: 10.1371/journal.pone.0193303 (PMC5832237; doi:10.1371/journal.pone.0193303)
Supplement: S6 Table — (DOCX) [file pone.0193303.s008.docx]

**Characteristics of included studies**：

*Liu 2017*

| methods | A prospective, randomized, single-blind study. |
| --- | --- |
| participants | age ≥18 years, elective cardiac surgery with cardiopulmonary bypass (CPB), admitted to inten- sive care unit (ICU) while intubated and ventilated, and lack of prior AF or flutter before receiving sedation in the ICU. |
| Interventions | randomized to receive either dexmedetomidine (0.2-1.5 μg/kg/h) or propofol (5-50 μg/kg/min)  open-label titrated  。 |
| outcomes | primary endpoint was the Sublingual microcirculation：  Atrial fibrillation occurred in 6 of 44 patients (13.6 %) in the dexmedetomidine group compared to 16 of 44 patients (36.4 %) in the propofol group (odds ratio = 0.28; 95 % confidence interval, 0.10, 0.80; P = 0.025).  B.The median (interquartile range) length of ICU stay in the dexmedetomidine group was significantly lower than in the propofol group (2.9 (2.4–3.5) vs 3.5 (2.7–4.5 days, P = 0.008), The incidence of hypotension was higher in the dexmedetomidine group than in the propofol group (25/44 (56.8 %) vs 13/44 (29.5 %); P = 0.017). |
| notes |  |

***Risk of bias***

| **Bias** | **Authors’ judgement** | **Support for judgement** |
| --- | --- | --- |
| Random sequence generation (selection bias) | Low risk | they were randomly assigned at a 1:1 ratio to receive sedation with either propofol or dexmede- tomidine according to the random number table. |
| Allocation concealment (selection bias) | unclear risk |  |
| Blinding of participants and personnel (performance bias) All outcomes | high risk | Open-lable.Can not be performed blinded. |
| Blinding of outcome assessment (detection bias)  All outcomes | Unclear risk |  |
| Incomplete outcome data (attrition bias) All outcomes | low risk | The proportion of the two groups is proportions |
| selective reporting (reporting bias) | Low risk | has protocol available，no unavailable data. |
