# Supplementary material for: Does dexmedetomidine have an antiarrhythmic effect on cardiac patients? A meta-analysis of randomized controlled trials
Source: PLoS One. 2018 Mar 1;13(3):e0193303. doi: 10.1371/journal.pone.0193303 (PMC5832237; doi:10.1371/journal.pone.0193303)
Supplement: S7 Table — (DOCX) [file pone.0193303.s009.docx]

**Characteristics of included studies**：

Ren 2013

| methods | Randomized controlled trial |
| --- | --- |
| participants | patients undergoing off-pump coronary artery bypass (OPCAB)《75 years old, 12 h of postoperative sedation and ventilation. |
| Interventions | dexmedetomidine versus propofol／Following the first vascular anastomosis grafting, the patients in group Dex received a continuous intravenous infu- sion of 0.2-0.5 μg/kg/h dexmedetomidine, until they were transferred to the Cardiac Surgery intensive care unit (ICU) for 12 h. The patients in group C were administered an intravenous infusion of 0.9% physiological saline, using identical infusion times and methods to group Dex. Following transferral to the Cardiac Surgery ICU, the patients received an intravenous infusion of 2-4 mg/kg/h propofol for sedation. |
| outcomes | 1. The blood pressure, heart rate , postoperative arrhythmic events, duration of mechanical ventilation and ICU residence time: were shorter than those in the control group (P<0.05). 2. AF: dexVS propofol (P<0.05). 3. SVT: dexVS propofol (P<0.05). 4. VT: dexVS propofol (P<0.05).   HR:control group：  DEX：  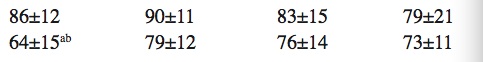 |
| notes | saline, propofol,(infusion) |

***Risk of bias***

| **Bias** | **Authors’ judgement** | **Support for judgement** |
| --- | --- | --- |
| Random sequence generation (selection bias) | unclear risk | Just said:randomly assigned ,no what kind of method. |
| Allocation concealment (selection bias) | Unclear risk | Not mentioned |
| Blinding of participants and personnel (performance bias) All outcomes | Unclear risk | Not mentioned |
| Blinding of outcome assessment (detection bias)  All outcomes | Unclear risk | Not mentioned |
| Incomplete outcome data (attrition bias) All outcomes | low risk | No incomplete data. |
| selective reporting (reporting bias) | High risk | No protocol available,but duration of mechanical ventilation and ICU residence time showed no data. |
