# Supplementary material for: Does dexmedetomidine have an antiarrhythmic effect on cardiac patients? A meta-analysis of randomized controlled trials
Source: PLoS One. 2018 Mar 1;13(3):e0193303. doi: 10.1371/journal.pone.0193303 (PMC5832237; doi:10.1371/journal.pone.0193303)
Supplement: S8 Table — (DOCX) [file pone.0193303.s010.docx]

**Characteristics of included studies**：

*Shehabi 2009*

| methods | a randomized, double-blinded, controlled clinical trial. |
| --- | --- |
| participants | Patients undergoing pump cardiac surgery, including coronary artery bypass grafts (CABG), valve surgery, combination CABG, and/or valve replacement proce- dures／**A total of 306 patients at least 60 yr old** |
| Interventions | dexmedetomidine versus morphine／a concentration of 0.1μg · kg-1 · ml-1 for dexmedetomidine and 10μg · kg-1 · ml- 1 for morphine, within 1 h of admission to the cardiothoracic inten- sive care unit (ICU), the study drug infusion commenced at 3 ml/h without a loading dose. Patients received an infusion of either dexmedetomidine (0.1– 0.7 􏰷g · kg􏰾 1 · ml􏰾1) or morphine (10–70 􏰷g · kg􏰾1 · ml􏰾1), which was titrated per prespecified protocol to maintain target se- dation and adequate analgesia. A propofol infusion and/or boluses were also given if deemed necessary by the medical team for rapid control of a hypertensive episode (systolic blood pressure 􏰿 160 mmHg) or un- planned awakening. drug infusion was continued until the re- moval of chest drains, when patient was ready to dis- charge from ICU, or for up to 48 h of mechanical venti- lation |
| outcomes | **Primary outcome was the prevalence of delirium measured daily** via **Confusion Assessment Method for intensive care. Secondary outcomes included ventilation time, additional seda- tion/analgesia, and hemodynamic and adverse effects.**:   1. Dexmedetomidine group showed no more benefits in sedation and pain levels, additional sedative/analgesic requirements, and extubation time;but more Incidence of Bradycardia:   D vs M: **Delirium incidence was comparable between dexmedetomidine 13 (8.6%) and morphine 22 (15.0%) (relative risk 0.571, 95% confidence interval [CI] 0.256 –1.099,** P 􏰹 **0.088),**  **extubation：more likely to be extubated earlier (relative risk 1.27, 95% CI 1.01–1.60,** P 􏰹 **0.040, log-rank** P ＝**0.036)**  **More bradycardia（16.45％ vs 6.12％，P = 0.006），Less incidence of hypotension** |
| notes | 306 eligible patients, each group 152 VS 147, 7 people out of Midway |

***Risk of bias***

| **Bias** | **Authors’ judgement** | **Support for judgement** |
| --- | --- | --- |
| Random sequence generation (selection bias) | Low risk | Patients were randomized via random com- puter-generated blocks of ten by the clinical trials phar- macist who also prepared study drug solution. |
| Allocation concealment (selection bias) | Unclear risk | Not mentioned |
| Blinding of participants and personnel (performance bias) All outcomes | low risk | All caregivers, including surgeons, anesthetists, and intensive care medical and nursing staff were blinded to the treatment given. |
| Blinding of outcome assessment (detection bias)  All outcomes | low risk | A reliable blind has been implemented, and the blind is unlikely to be broken |
| Incomplete outcome data (attrition bias) All outcomes | low risk | has incomplete data, but has exact explaint and:As surgery was a critical entry point to the study protocol, data were assessed by using a modified inten- tion-to-treat (ITT) population with primary analysis per- formed on patients |
| selective reporting (reporting bias) | Low risk | No protocol available |
