# Supplementary material for: Does dexmedetomidine have an antiarrhythmic effect on cardiac patients? A meta-analysis of randomized controlled trials
Source: PLoS One. 2018 Mar 1;13(3):e0193303. doi: 10.1371/journal.pone.0193303 (PMC5832237; doi:10.1371/journal.pone.0193303)
Supplement: S9 Table — (DOCX) [file pone.0193303.s011.docx]

**Characteristics of included studies**：

Göksedef 2013

| methods | Randomized controlled trial |
| --- | --- |
| participants | consecutive 100 patients who had previously undergone coronary artery bypass graft (CABG) surgery using a heart-lung machine.／for a total of 86 patients (mean age 61.2±11.2; range, 34-78 years). |
| Interventions | dexmedetomidine versus placebo／the subjects were randomized into a DEX infusion group (n=49) and a placebo group (n=37) , Infusion solutions were administered at a rate of 0.04 μg/kg/hour postoperatively in the ICU. in a maximum 24-hour period. In addition, all patients received intravenous morphine (2-4 mg) every 4-6 hours on an as needed basis. |
| outcomes | to investigate possible effects of dexmedetomidine on renal functions following coronary artery bypass graft (CABG) surgery.:   1. no significant differences existed in the groups concerning atrial fibrillation (AF) (p= 0.444);   extubation time(P>0.05); Intensive care unit time (hours) (P>0.05); Hospital stay (days) (P>0.05)  No significant differences between the two groups for mortality (0.382) (P>0.05) |
| notes | Excluded 14 patients(The proportion of the two groups is disproportions) |

***Risk of bias***

| **Bias** | **Authors’ judgement** | **Support for judgement** |
| --- | --- | --- |
| Random sequence generation (selection bias) | Low risk | Patients were randomized to either dexmedetomidine infusion or placebo. Permuted blocks of four method was used to randomize patients. |
| Allocation concealment (selection bias) | Unclear risk | Not mentioned |
| Blinding of participants and personnel (performance bias) All outcomes | low risk | we undertook a double-blind study featuring 100 consecutive patients。The DEX infusions were prepared according to the randomization list by a single nurse outside the ICU, and the list was kept secret until the end of the study. |
| Blinding of outcome assessment (detection bias)  All outcomes | Unclear risk | Not mentioned |
| Incomplete outcome data (attrition bias) All outcomes | high risk | has incomplete data. The proportion of the two groups is disproportions |
| selective reporting (reporting bias) | Low risk | No protocol available， |
